# Investigating the Synergistic Effect of Decoration and Doping in Silver/Strontium Titanate for Air Remediation

**DOI:** 10.3390/nano14201663

**Published:** 2024-10-16

**Authors:** Marcela Frías Ordóñez, Elisabetta Sacco, Marco Scavini, Giuseppina Cerrato, Alessia Giordana, Ermelinda Falletta, Claudia Letizia Bianchi

**Affiliations:** 1 Department of Chemistry, University of Milan, Via Golgi 19, 20133 Milano, Italy; marcela.frias@unimi.it (M.F.O.); elisabetta.sacco@unimi.it (E.S.); marco.scavini@unimi.it (M.S.); claudia.bianchi@unimi.it (C.L.B.); 2 Consorzio Interuniversitario Nazionale per la Scienza e Tecnologia dei Materiali (INSTM), Via Giusti 9, 50121 Florence, Italy; giuseppina.cerrato@unito.it (G.C.); alessia.giordana@unito.it (A.G.); 3 Department of Chemistry, University of Turin, Via Giuria 7, 10125 Turin, Italy

**Keywords:** Ag-SrTiO_3_ materials, NOx photodegradation, effect of ethanol, thermal stability

## Abstract

Strontium titanate (STO) and its variants have emerged as leading materials in photocatalysis, particularly for degrading nitrogen oxides (NOx), due to their non-toxic nature, structural adaptability, and exceptional thermal stability. Although the one-pot sol-gel method leads to high-quality photocatalysts, areas remain for improvement. This study examines the impact of ethanol as a cosolvent in STO synthesis, focusing on optimizing the water-to-ethanol volume ratio. The findings reveal that a 1:3 ratio significantly enhances macropore formation and photocatalytic efficiency, achieving 42% NOx degradation under LED within three hours. Furthermore, incorporating 8.0 wt.% Ag into STO substantially improves visible light absorption and enables complete NOx elimination, thanks to enhanced charge separation and localized surface plasmon resonance. Even at high temperatures (1100 °C), the Ag-STO photocatalyst maintains partial activity, despite exceeding silver’s melting point. These results highlight the potential of STO-based materials for industrial applications, positioning them as a promising solution for effective NOx mitigation.

## 1. Introduction

Air pollution poses a dual threat to human health and environmental sustainability worldwide. The challenge arises from the broad and pervasive presence of noxious air contaminants from various sources. Industrial activities, transport, and power generation are significant contributors that discharge a range of pollutants, including particulate matter (PM), nitrogen oxides (NOx), sulfur oxides (SOx), and volatile organic compounds (VOCs). Prolonged exposure to these pollutants has been linked to respiratory, cardiovascular, and immune-related illnesses, as well as millions of premature deaths annually [1,2].

Nitrogen oxides (NO, NO_2_, NOx) are noxious gases principally generated by combustion processes in vehicles and industry and have an injurious impact on the atmosphere, human health, and plants. NOx gases are associated with acid rain formation, photochemical smog, depletion of the ozone layer, and ecological toxification [3]. Various materials have been developed, investigated, and optimized for their removal, including metal–organic frameworks (MOFs), activated carbon, zeolites, and functionalized metal oxides [4]. Selective catalytic reduction (SCR) and selective non-catalytic reduction (SNCR) are widely used abatement strategies due to their high efficiency, versatility, and suitability for industrial applications [3]. In contrast, photocatalysis is an innovative and sustainable technique with considerable potential for air purification, including nitrogen oxide degradation [5]. The use of natural or artificial light, low-cost material, and ambient working conditions are some of the numerous advantages of photocatalytic abatement. Titanium dioxide (TiO_2_) has emerged as a leader in NOx photocatalytic abatement. This is due to its exceptional photoactivity under UV illumination (wavelength < 400 nm) as a result of its wide energy band gap (3.2 eV) [6]. However, given that individuals spend approximately 80–90% of their time indoors, whether at home, in the workplace, or at school, the broad energy band gap of TiO_2_ becomes a limiting factor, as it does not absorb the visible region [7]. Additionally, the irreversible phase transition (anatase → rutile) that occurs above 600 °C restricts the industrial application of TiO_2_ in specific sectors [8]. Based on these premises, the scientific community is exploring alternatives with greater thermal stability, such as perovskites.

Perovskite materials have undergone extensive study since the middle of the 20th century due to their extraordinary properties, including dielectric, piezoelectric, electronic conductivity, and thermal and optical features [9]. These materials have particularly interested researchers because of their potential to address energy and environmental challenges [10,11]. Strontium titanate (SrTiO_3_) is a well-known semiconductor material with a cubic-like structure. It is sought after due to its high thermal stability, corrosion resistance, and energy band gap similar to TiO_2_ [12,13]. Its excellent photocatalytic performance under UV light is also noteworthy. Therefore, to enhance SrTiO_3_′s photo-response within the visible light spectrum, implementing a few techniques can be considered. In this work, the surface of SrTiO_3_ was functionalized with Ag nanoparticles, utilizing the localized surface plasmon resonance (LSPR) effect, which enhances absorption within the visible light range [14]. The Schottky barrier formed between Ag and the semiconductor promotes efficient electron capture, thereby reducing the recombination rate between electrons and holes. Another benefit to using Ag is related to its antimicrobial properties [15]. Ag-modified semiconductors and, in particular, Ag-modified SrTiO_3_ have already been studied in the literature. Ag-SrTiO_3_ nanocomposites (wt.% Ag = 0.1, 0.25, 0.5%, and 1%) were synthesized via a one-pot solvothermal method (1:6 water/ethanol volume ratio) at 350 °C in an atmosphere of Ar by Zhang et al. [16]. The study revealed that adding 0.5 wt.% Ag nanoparticles to SrTiO_3_ nanocubes (about 40 nm) effectively doubled STO photoactivity. This resulted in 30% of NO removal under visible light irradiation within 30 min. The authors attributed the improvement in photocatalytic activity to the light harvest stemming from plasmon resonance of 10–20 nm Ag nanoparticles. Additionally, the Ag-STO heterostructure reinforced the electron–hole separation in the interface.

On the contrary, Ma et al. [17] utilized a multi-step synthesis approach for Ag-SrTiO_3_ materials, involving a hydrothermal method (at 180 °C) followed by photochemical reduction. The authors established that the 15 mol.%Ag-SrTiO_3_ photocatalyst, incorporating oxygen vacancies, manifested the utmost removal efficiency (70% of NO photodegradation) under visible light within 30 min. Similar to the results of Zhang et al. [16], the visible light response was associated with integrating Ag nanoparticles, which Ma et al. [17] demonstrated was more pronounced with increasing amounts of Ag. Additionally, our previous research aimed at the preparation of a 5 wt.% Ag-modified SrTiO_3_ photocatalyst through a water-based one-pot method with heat treatment at 900 °C. Using this approach, the SrTiO_3_ ceramic substance was dually altered inside the lattice (Ag^+^ doping) and its surface (Ag nanoparticles’ decoration). This led to a comprehensive NOx photodegradation in 3 h using LED, causing a fourfold surge in photocatalytic effectiveness compared to pristine SrTiO_3_ [18]_._

The synthesis parameters, including solvent and calcination temperatures, significantly affect the photocatalytic properties [19], but no studies have addressed the impact of solvent on Ag-SrTiO_3_ material preparation and its effect on photocatalytic activity. Moreover, no research has ever been conducted on the stability of such materials when exposed to the severe scenarios (very high temperatures) expected in some industrial applications. This study used a one-pot sol-gel method with a water/ethanol system for fabricating SrTiO_3_ photocatalysts. The volume ratio between both solvents was evaluated and optimized for the materials’ synthesis, and their photocatalytic efficiencies for NOx abatement under LED lighting were assessed. We found that the 1:3 water/ethanol volume ratio resulted in macropores that significantly increased the photocatalytic activity. Ag-SrTiO_3_ materials were synthesized with varying weight ratios, calcined at 900 °C, and subsequently evaluated for photoactivity. The most effective material underwent rigorous investigation and demonstrated high stability for up to ten runs. However, an assessment of the thermal stability of these materials is currently lacking. Therefore, the best photocatalyst was exposed to a temperature exceeding silver’s melting point (961 °C), specifically at 1100 °C. This evaluation aimed to examine the effects of heat treatment conditions on the catalyst’s structural, morphological, and photocatalytic characteristics, specifically regarding potential real industrial applications.

## 2. Materials and Methods

### 2.1. Materials

Strontium acetate (C_4_H_6_O_4_Sr), titanium(IV) isopropoxide (C_12_H_28_O_4_Ti), silver nitrate (AgNO_3_), citric acid (C_6_H_8_O_7_), and ethanol absolute (≥99.0%) were purchased from Merck (Darmstadt, Germany). They were analytical grade and used without further purification. 

### 2.2. Synthesis of SrTiO_3_ in Ethanol/Water (1:3 and 1:5 Volume Ratio) 

Bare SrTiO_3_ (STO) perovskites were prepared via the one-pot sol-gel method. The following quantities are estimated for 5 g of the final product. Briefly, solution A was prepared by adding titanium(IV) isopropoxide (TTIP, 27 mmol) dropwise into ethanol (100 mL) under vigorous stirring (300 rpm) for 15 min. Ethanol was used as a green solvent to avoid the immediate hydrolysis of TTIP to TiO_2_ in aqueous solutions. Then, citric acid (54 mmol) was added in a molar ratio of 2:1 with respect to Ti^4+^ ions. On the other hand, a solution B was prepared by dissolving a stoichiometric amount of strontium acetate (27 mmol) in the minimum amount of water (20 mL). The Ti and Sr precursors were taken in a 1:1 molar ratio. Afterward, citric acid (54 mmol) was added in a molar ratio of 2:1 with respect to Sr^2+^ ions. The two solutions were mixed at room temperature for 3 h and then evaporated using an oil bath at 110 °C. Once the colorless gel was obtained, it was transferred into a crucible and dried for 14 h at 60 °C followed by 8 h at 120 °C. Finally, the samples were calcined at 900 °C, adopting the following heating ramp: 250 °C for 1 min (0.5 °C/min) and 900 °C for 1 h (2.5 °C/min). 

To investigate the effect of the concentration of ethanol amount into the solvent used during the materials synthesis on the photocatalysts’ structure and their photocatalytic activity, different volume ratios of water/ethanol (1:3 and 1:5) were employed throughout the above procedure. The synthesized samples will be named STO (1:3) and STO (1:5) hereafter. The photocatalytic performance of both samples was evaluated (paragraph 2.3), and higher efficiency was observed for the STO (1:3) sample. For this reason, this material was selected for further Ag modification.

### 2.3. Synthesis of Ag-STO (wt% Ag = 1.0, 2.5, 4.0, 5.8, 8.0)

STO (1:3) demonstrated a good photocatalytic performance towards NOx degradation from the previous procedure. Therefore, the synthesis of Ag-SrTiO_3_ materials was performed considering a water/ethanol volume ratio of 1:3. The amount of reactant used was determined stoichiometrically, following the hypothesis that silver ions are introduced into the strontium sublattice. Therefore, the formula of the desired compound will be Sr_1−x_Ag_x_TiO_3_. In this context, the amount of silver was assessed (wt.% Ag = 1.0, 2.5, 4.0, 5.8, 8.0), and the corresponding molar ratios of individual elements are reported in Table S1. The following quantities are estimated for 5 g of the 8.0 wt.% Ag-STO photocatalyst. Firstly, solution A was prepared by adding TTIP (27 mmol) into ethanol (60 mL) under vigorous stirring (300 rpm). Then, citric acid (54 mmol) was added in a molar ratio of 2:1 with respect to Ti^4+^ ions. On the other hand, solution B was prepared by dissolving a stoichiometric amount of silver nitrate (4 mmol) and strontium acetate (23 mmol) in the minimum amount of deionized water (20 mL). Then, citric acid (54 mol) was added in a molar ratio of 2:1 for the sum of Ag^+^ and Sr^2+^ ions. The synthesis of the material proceeds as reported above. Similarly, the Ag-SrTiO_3_ photocatalysts with different weight percentages of Ag (1.0%, 2.5%, 4.0%, 5.8%, and 8.0%) were synthesized, and the samples were labeled as Xwt.% Ag-STO, where X corresponds to the Ag percentage. 

### 2.4. Heat Treatment Conditions of 8.0%Ag-STO Photocatalyst

In this study, the best-performing photocatalyst, 8.0 wt.%Ag-STO, was calcined at 1100 °C above silver’s melting point (T_m_ = 961 °C), as listed in Table 1, to stress and test the thermal stability of the material, verify the impact on the photocatalytic activity, and identify the more appropriate heat treatment conditions. 

### 2.5. Characterization 

The X-ray diffraction (XRD) patterns of all the samples were carried out at room temperature in the 10 ≤ 2θ ≤ 80° range (Δ2θ = 0.02°) with a Rigaku MiniFlex 600 diffractometer (Rigaku, Tokyo, Japan), equipped with a Cu anode, a Ni filter to remove the Cu-K_β_ radiation, and a D/teX Ultra detector. Each pattern took 30 min. 

To better characterize the Ag phase(s), synchrotron radiation high-resolution powder diffraction patterns were collected on the Ag-STO photocatalysts with 5.8% and 8.0% Ag at the ID22 beamline of the ESRF [20] during the experiment hc5201. The powder samples were loaded into 1.0 mm diameter Kapton^®^ capillaries and measured using an X-ray wavelength λ = 0.332415(1) Å and a 13-element detector array in the 0° ≤ 2θ ≤ 42° interval (Q_*max*_ = 13.55 Å^−1^). Also, a LaB_6_ standard has been measured in the same experimental conditions to obtain the instrumental resolution function.

All the XRD experimental patterns were analyzed using the Rietveld analysis, which utilized the GSAS software suite of programs [21] and its graphical interface, EXPGUI [22]. In the last refinements, the cell constants and one average thermal parameter were varied to the phase fractions, the background (through the Chebyshev polynomial), and the line profile.

The synchrotron radiation measurements have also been used to calculate the crystallite dimension of Ag by means of the Williamson–Hall method [23]. After deconvolution of the instrumental contribution, the Gaussian and Lorentzian contributions to peaks FWHM, as computed during the Rietveld refinements (program widplot in GSAS), were used to calculate the integral breath β_J_ of each peak j of Ag phases centered in 2θ_J_ position; then, the (2θ_J_, β_J_) were plotted as

β_J_ cos(θ_J_) = 4ε sin(θ_J_) + λ/D_V_ (1)

where ε is the strain parameter, λ is the experimental wavelength, and D_V_ is the volume-weighted crystallite dimension. 

Field emission scanning electron microscopy (FE-SEM) and HR-TEM evaluated the materials’ morphology and elemental composition. The FE-SEM images were collected using a scanning electron microscope operating with a field emission source ((model TESCAN S9000G, (Brno, Czech Republic); source: Schottky type FEG; resolution: 0.7 nm at 15 keV (in in-beam SE mode) and equipped with EDS Oxford Ultim Max (operated with Aztec software 6.0). HR-TEM images were obtained by means of a Jeol 3010-UHR instrument (Tokyo, Japan ) operating at 300 kV (LaB6 filament). Samples were dry-dispersed onto Cu holey grids coated with pyrolytic carbon before inspection. The specific surface area (SA) and porosity (pore volume, pore size, and pore size distribution) were determined by N_2_ adsorption/desorption isotherms using the Coulter SA3100 instrument (Beckman Coulter, Brea, CA, USA). The samples were pre-treated at 150 °C for 4 h under vacuum to remove adsorbed foreign species. The specific surface areas of the samples were calculated with the Brunauer–Emmett–Teller (BET) equation (two parameters, 0.05 < p/p_0_ < 0.3); whereas, the pore size and pore volume were estimated by using the Barrett–Joyner–Halenda (BJH) model from the desorption isotherm (0.3 < p/p_0_ < 0.95). Diffuse-reflectance UV-vis (UV-DRS) spectra were recorded in the 200 nm to 800 nm range on a Perkin Elmer Lambda 750s UV-Vis spectrophotometer (PerkinElmer, Inc. Waltham, MA, USA) with an integrating sphere assembly using BaSO_4_ as a reference standard. The spectra were obtained in reflectance mode, and the energy band gap was estimated by the Tauc plot of the Kubelka–Munk function (Equation (2)):

(2) $$F\left(R\right)=\frac{{\left(1-R_{\infty }\right)}^{2}}{2R_{\infty }}$$

where R∞ (R_sample_/R_BaSO_4__) corresponds to the sample reflectance, and F(R) is the Kubelka-Munk function [24]. In Equation (2), *hv* is the incident photon energy, and n is the sample transition, which is 2, since STO is an indirect band gap semiconductor [25].

### 2.6. Photocatalytic NO_x_ Tests

**Figure 1:**
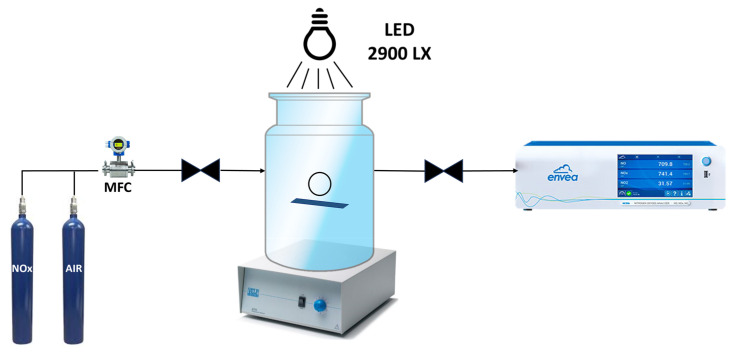
Schematic representation of the experimental set-up employed for the NOx photodegradation.

All STO and Ag-STO materials’ photocatalytic activity for NOx degradation was assessed under LED irradiation. The sample was prepared by weighing 50 mg of each photocatalyst and suspending it in 5 mL of isopropanol, assisted by an ultrasonic bath. Then, a thin film of the compound was drop-cast onto a 3.3 cm × 1.5 cm glass plate placed inside a 20 L Pyrex cylindrical batch reactor after the solvent evaporated. The tests were conducted at room temperature with an initial NOx concentration of 500 ± 50 ppb (composed of 0.625% of NO_2_ and 0.125% of NO, diluted with air) under LED irradiation (Philips Lamp—350 mA, 9–48 V, 16.8 W, 400–700 nm) for 3 h, yielding an intensity of 2900 lx on the photocatalyst surface (Figure 1).

Vigorous stirring applied to the base of the reactor ensured turbulence inside. The NO_x_ concentration was monitored by a chemiluminescence analyzer (ENVEA AC32e; Envea, Poissy, France) directly connected to the reactor after 30, 60, and 180 min. Finally, photolysis tests showed 7% of pollutant degradation after 3 h of light irradiation. The reusability and stability potential of the most performing photocatalyst was investigated for 10 cycles under the same experimental conditions without any post-treatment of the photocatalyst.

At the end of the last cycle, the photocatalyst was recovered and subjected to three consecutive washes with small aliquots of deionized water (2 mL) at 50 °C under sonication.

Subsequently, the aliquots were analyzed by a colorimetric nitrate test (MQuant, Merck; Darmstadt, Germany) to verify that the oxidation of NOx to nitrate had occurred.

## 3. Results and Discussion

### 3.1. Materials’ Characterization

**Figure 2:**
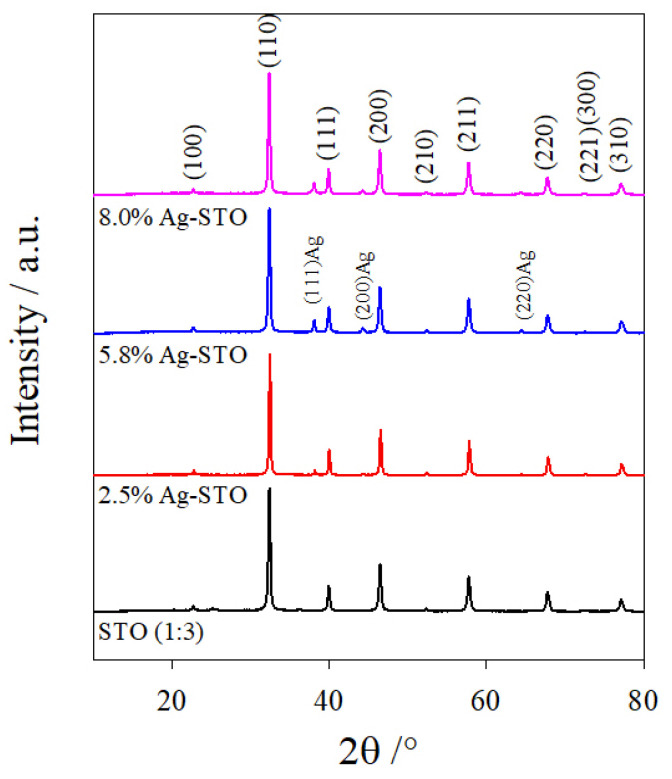
Experimental patterns of STO (black curve), 2.5% Ag-STO (red curve), 5.8% Ag-STO (blue curve), and 8.0% Ag-STO (pink curve).

Figure 2 shows the XRD experimental patterns collected on the selected investigated samples. The data were analyzed using the Rietveld method, as described in the experimental section. Figure S1 shows some examples of refinements, while the computed structural parameters on all the samples heat-treated at 900 °C are listed in Table S2. Sample STO (1:3) was composed of two phases: the cubic perovskite phase of SrTiO_3_, space group $Pm\bar{3}m$ [26,27], and anatase TiO_2_ (space group $I4_{1}/amd$) [28], as an impurity phase (weight percentage wt.% ≈ 5.5%). In the patterns of the Ag-containing samples, the second phase suddenly disappeared and an additional cubic Ag phase progressively grew up (space group $Fm\bar{3}m$) [29] upon increasing the amount of dopant. However, the refined metal Ag weight percentage was always lower than the nominal Ag content, fixed by the synthesis. This mismatch could have a different origin. On the one hand, the missing Ag could have been incorporated in the strontium titanate structure, as an example, substituting a Sr ion; on the other hand, the quality of XRD patterns collected with the Rigaku MiniFlex 600 diffractometer (Rigaku, Tokyo, Japan) does not allow for accurate quantifying of the Ag phase due to its small weight percentage and probable nanosize.

For this reason, the samples containing the highest Ag amount (5.8 wt.% and 8.0 wt.% Ag-STO) were also measured at the high-resolution ID22 beamline.

The possibility that Ag substitutes Sr in the perovskite structure was neglected in the first Rietveld refinements. The theoretical atomic ratio fixed by the synthesis is 5.8 wt.% and 8.0 wt.% Ag-STO samples are Sr: Ag: Ti = 0.89:0.1:1.00 and 0.86:0.14:1.00, respectively. The multiphase fits involved only the Ag metal phase and perovskite phases with the nominal composition of Sr_0.90_TiO_3_ (for 5.8 wt.% Ag-STO) and Sr_0.86_TiO_3_ (for 8.0 wt.% Ag-STO). The quantitative analysis derived from the Rietveld refinements brought in both cases an Ag deficiency with respect to the expected ratios: Sr: Ag: Ti = 0.90:0.08:1.00 for 5.8 wt.% Ag-STO sample and Sr: Ag: Ti = 0.90:0.09:1.00 for 8.0 wt.% Ag-STO.

Again, the Ag deficiency detected by the analysis could have two origins: (A) part of the Ag species could not form (nano)crystalline phases but be included in small aperiodic clusters that do not introduce Bragg peaks in the XRD pattern, or (B) the missing silver could substitute Sr in the perovskite structure. Starting from the stoichiometry fixed by the synthesis and considering the phase fractions of the perovskite and metal Ag phases resulting from the previous fits, in the new refinements, perovskite phases of composition Sr_0.90_Ag_0.02_TiO_3_ and Sr_0.86_Ag_0.05_TiO_3_ for the 5.8 wt.% Ag-STO and 8.0 wt.% Ag-STO cases were adopted, respectively. In both cases, the new models improved the goodness of fit χ^2^ = 8.17 instead of 10.42 for 5.8 wt.% Ag-STO and χ^2^ = 9.63 instead of 16.07 for 8.0 wt.% Ag-STO. This result suggests that a minority part of the Ag (20% for 5.8 wt.% Ag-STO and 35% for 8.0 wt.% Ag-STO of the total amount) enters the perovskite structure. In fact, if scenario A was correct, imposing the partial occupation of Ag on the Sr site would have worsened the fit quality.

**Figure 3:**
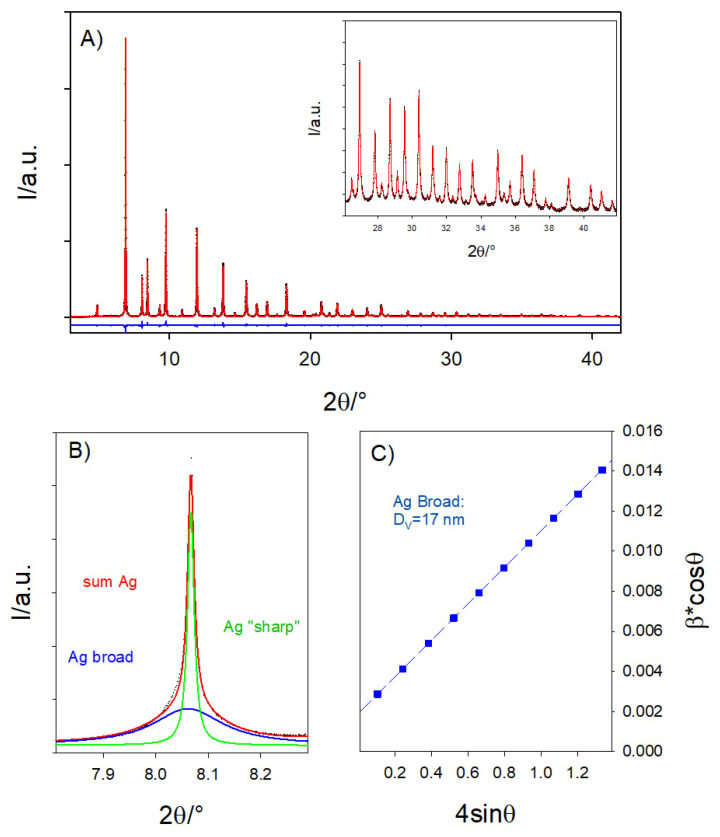
Panel (**A**): Complete experimental XRD pattern of 8.0 wt.% Ag-STO sample (black symbol) together with its Rietveld refinement (red curve) and the difference curve (blue curve). The inset highlights the high-angle data and refined curve. Panel (**B**): Details of the same pattern corresponding to the (111) reflection of Ag phase(s). Black symbols are the experimental data; blue and green curves show the contribution of “Ag-broad” and “Ag-sharp” phases to the peak while the red curve is their sum. See the main text for details. Panel (**C**): Williamson–Hall plot for the Ag-broad phase.

Panel A of Figure 3 shows, as an example, the Rietveld refinement for the 8.0 wt.% Ag-STO sample. In Figure 3, the experimental XRD patterns, the fitted curve, and the residuals are depicted as black symbols and red and blue curves, respectively. The refined parameters are reported in Table 2. The 8.0 wt.% Ag-STO pattern evidences a very small amount of TiO_2_ (wt.% ≈ 0.1) in rutile form [28].

Focusing now on the Ag metal phases, Figure 3B shows a small 2θ range of the same pattern, where the most intense diffraction peak of the metal Ag phase is located, that is, the (111) reflection. The broad tails of the peak suddenly sharpen upon reaching their maximum intensity. This is a fingerprint of the bimodal distribution of the crystal size, testifying to the presence of both Ag nano- and well-growth crystals. For this reason, besides the SrTiO_3_ cubic one in the final refinements, two different Ag phases have been considered, which will be called “Ag-broad” and “Ag-sharp”. It is essential to underline that Ag-broad and Ag-sharp must not be considered different from the thermodynamic point of view.

The sum of the two phases brings ≈5% and ≈6% metal Ag weight percentages for 5.8 wt.% Ag-STO and 8.0 wt.% Ag-STO samples, respectively. As computed by the Williamson–Hall method, the crystal dimension for the Ag-broad phase is ≈8 and ≈17 nm for 5.8 wt.% Ag-STO and 8.0 wt.% Ag-STO, respectively.

**Figure 4:**
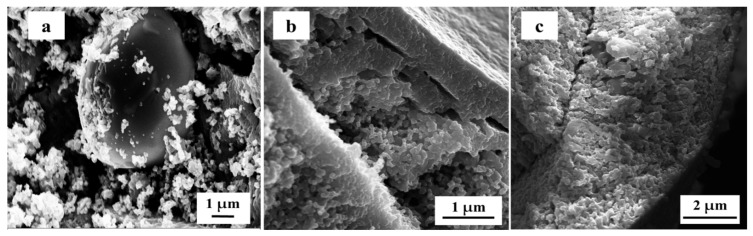
FE-SEM images of 8 wt.% Ag-STO_1100C with (**a**) 2 h, (**b**) 10 h, and (**c**) 15 h of heat treatment.

Focusing on the role of the heat treatment, the XRD patterns performed on the 8.0 wt.% Ag-STO material exposed to the highest temperature (1100 °C) revealed a decrease in the intensity of both the Ag peaks and the phase fraction of the metallic Ag phase, as computed by the Rietveld refinements of the experimental pattern (see Table S3). Since, as it is known, the melting point of Ag is 961.8 °C, exceeding this temperature leads to a negative effect on the Ag distribution, probably causing partial metal melting, aggregation, and/or diffusion in the deeper layers of the catalyst, as confirmed by the FE-SEM and EDX analyses (Figure 4 and Figures S3–S5, respectively).

However, it is interesting to observe that the intensity of the Ag peaks in the XRD patterns is related not only to the change in temperature (from 900 °C to 1000 °C and 1100 °C) but also to the residence time at the highest temperature. In particular, when the sample is annealed at 1100 °C for one night, the weight percentage of the metallic Ag phase is 0.027(1), while it passes to 0.037(1) and 0.042(1) when the residence time at 1100 °C is reduced to 10 and 2 h, respectively (see Table S3). Figure S2 shows details of the XRD patterns corresponding to the most intense metallic Ag peak after suitable normalizations. It is possible that by increasing the time spent at the highest temperature, the melted Ag particles can change their microstructure to be included in small aperiodic clusters that do not introduce Bragg peaks in the XRD pattern, or gradually and slowly penetrate the bulk, and eventually be incorporated in the STO structure. In accordance with the second scenario, as shown in Table S2, increasing the annealing time at 1100 °C, the lattice parameter of the STO phase increases too, passing from 3.90645(6) Å to 3.90698(6) Å.

The XRD investigation demonstrated that, despite Sr and Ag being introduced in stoichiometric amounts assuming that silver entered the crystal lattice in the Sr site, doping alone never occurs in all cases of the different Ag loads studied. However, a dual modification is obtained, observing both doping and decoration, as confirmed by FE-SEM and HR-TEM investigations as described below ).

**Figure 5:**
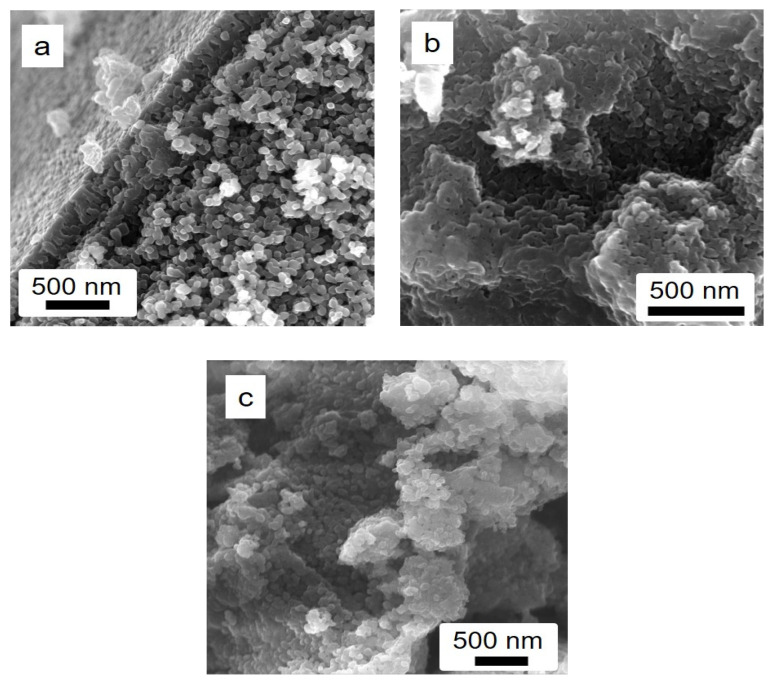
FE-SEM images of (**a**) STO (1:3), (**b**) STO (1:5), and (**c**) 8 wt.% Ag-STO.

In order to investigate the effect of the ethanol amount into the solvent used during the materials’ synthesis on the morphological characteristics, all the STO-based photocatalysts were characterized by field emission scanning electron microscopy (FE-SEM). Figure 5a,b shows that both STO (1:3) and STO (1:5) materials exhibit a cubic-like morphology with particles of ca. 50 nm average dimension and an entirely different level of agglomeration [18].

**Figure 6:**
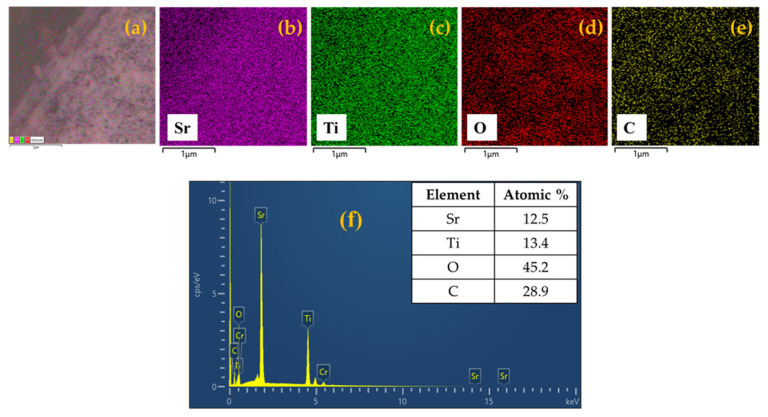
SEM image (**a**), elemental mapping of strontium (**b**), titanium (**c**), oxygen (**d**), carbon (**e**), and EDX spectrum (**f**) of STO (1:3) photocatalyst.

This feature is much more pronounced in the case of the STO (1:5) sample (see section b in Figure 5), and it might be due to the different ratios of water/ethanol added in the synthetic procedure. When Ag species are added (see Figure 5c, which refers to the 8.0 wt.% of Ag), the general morphology of the STO system remains almost unaltered, retaining the average dimensions of the crystallites as well. However, the presence of Ag species is hardly evidenced in the FESEM images, even if they have been obtained at high magnification, indicating that the average size of the Ag particles is of tiny dimensions and possibly in the nanometric range (and out of the resolution of the FESEM technique), as demonstrated by the XRD investigations. In order to confirm that the Ag species are present, EDX analyses were performed for all the samples in both the absence and the presence of Ag species. The first experimental result of both pure STO materials indicates that Sr, Ti, and O elements are present in the stoichiometric proportion and are found to be homogeneously distributed (see the EDX analyses and elemental mapping reported in Figure 6 and Figure S6).

**Figure 7:**
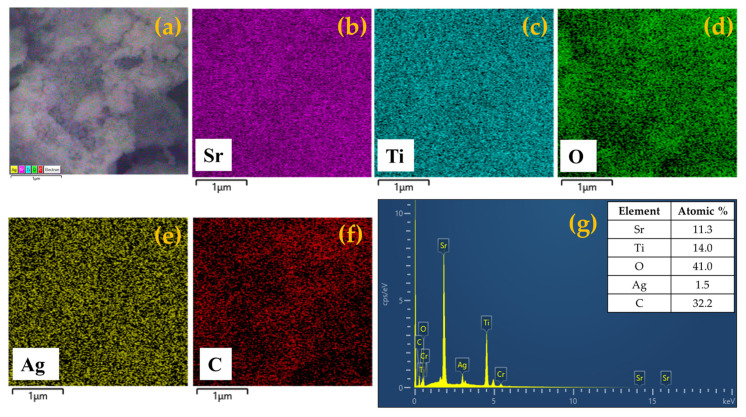
SEM image (**a**), elemental mapping of strontium (**b**), titanium (**c**), oxygen (**d**), silver (**e**), carbon (**f**), and EDX spectrum (**g**) of 8 wt.% Ag-STO photocatalyst.

In the case of the Ag-containing sample, the EDX spectrum confirms the presence of this species (ca. 1.5 wt.%); additionally, all the others and the elemental mapping provide evidence of its location in the material, with a good distribution, without any Ag NP agglomeration (Figure 7).

The thermal treatment’s effect on morphology and silver distribution of 8.0 wt.% Ag-STO material was also investigated through FESEM and EDX analyses (see Figure 4 and Figures S3–S5).

Firstly, the increase of 200 °C in the heat treatment (from 900 °C to 1100 °C) led to a certain aggregation of STO particles, producing much larger particles (>200 nm): only 2 h of this heat treatment provided a massive aggregation (see Figure 4a). As expected, longer times required to reach 1100 °C (10 and 15 h, respectively) promoted an advanced level of particle aggregation, where the cubic-like morphology of STO particles was almost totally lost (Figure 4b,c). Regarding both the distribution and size of Ag particles onto the STO surface, high temperatures and long times (1100 °C and 15 h) resulted in a thorough loss of Ag at the surface (0.4 at. %) compared to the starting 8 wt.% Ag-STO sample calcined at 900 °C (*ca.* 1.5 at.% Ag), according to the XRD investigations (Figure S2). In contrast, the presence of Ag micro-particles (>5 µm) in ca. 6 at. % Ag amount at the surface of the photocatalyst is evidenced for shorter times (2 and 10 h) of thermal treatment at 1100 °C: this suggests a possible displacement, accumulation, and odd distribution, as can be seen in Figure 4 and Figures S3–S5.

**Figure 8:**
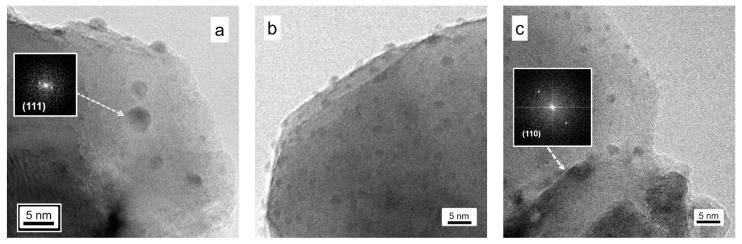
HR-TEM images of 8 wt.% Ag-STO calcined at increasing temperatures and for different times. Section (**a**): sample calcined at 900 °C; Section (**b**): sample calcined at 1100 °C for 2 h; Section (**c**): sample calcined at 1100 °C for 15 h.

HR-TEM has been reported to have indications about the morphology and dimensions of the Ag species, and the relevant results are reported in Figure 8, in which it is possible to single out the Ag effective location.

Above all, it is evident that the main STO features are totally retained even in the presence of Ag addition, as already reported in the literature [18] and observed by the FE-SEM studies. As for the Ag species, they are clearly observable in the form of tiny nanoparticles, whose dimensions lie in the 1–4 nm range and exhibit (in the same specific experimental situations) a hexagonal shape. The simulated electron diffraction pattern, obtained by means of the FFT on the fringe pattern reported in Figure 8, indicates that the main crystallographic plane of these Ag NPs belongs to the (111) family of metallic silver with d(hkl) = 0.23_6_ nm along the ICDD card n. 00-004-0783.

Employing HR-TEM for inspecting the high-temperature treated materials in order to confirm the indications coming from the FESEM analyses (see the relevant discussion previously reported), it can be claimed that the STO material suffered an aggregation without bringing about a loss in the high crystallinity of the samples. This is confirmed even after 15 h of thermal treatment (see Figure 8c), and characteristic fringe patterns belonging to STO are clearly observable due to the (110) family of planes with d(hkl) = 0.27_6_ nm) along the ICDD card n. 00-035-0734 (also confirmed by the FFT calculation, simulating the electron diffraction, reported in the inset to Figure 8c). As for the morphology and location of the Ag NP species, it is evident that these species are still present (as already singled out by the FESEM analyses), with very tiny dimensions (less than 2 nm in diameter), and are not always located on top of the STO material, but probably under the surface, even if quite close to it.

**Figure 9:**
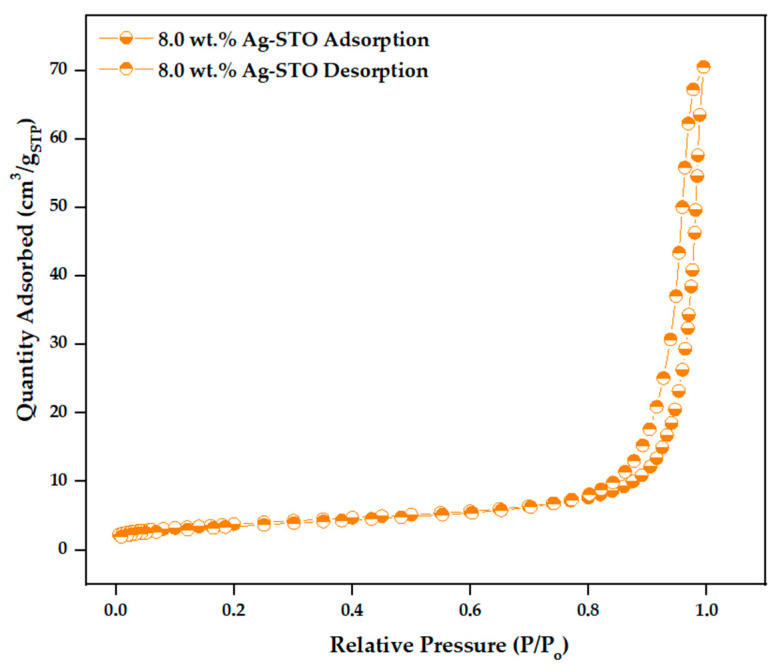
N_2_ adsorption/desorption isotherms at −196 °C.

Furthermore, the correlation between the surface properties and photocatalytic performance of the materials is significant [30,31]. N_2_ physisorption isotherms were collected to investigate surface area values and porosity of samples (Figure 9 and Figure S7).

In the literature, isotherms of similar samples are considered type IV, according to the IUPAC classification [32]. However, based on our knowledge, they fall in the type II category, as they do not reach a plateau at a high relative pressure (Figure 9 and Figure S7). This profile corresponds to non-porous materials. A slight type H1- hysteresis loop indicates mesopores extending into the macroporous region. Generally, both studied STO samples were described by a specific surface area value of 23 m^2^/g. The successive introduction of Ag (bulk + surface) caused a decrease in surface area, which was even more pronounced by increasing the calcination temperature and time. The effect of calcination parameters was also detectable in the pore volume. Also, in this case, the increasing calcination temperature reflects a decrease in the pore volume (Table 3), which could be related to the partial sintering of particles at high temperatures, followed by the collapse of pores [33].

**Figure 10:**
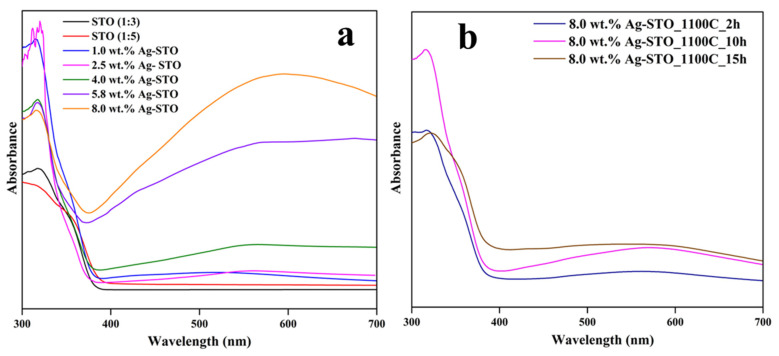
UV-DRS spectra of (**a**) Ag-STO photocatalysts at 900 °C and (**b**) 8 wt.% Ag-STO_1100C treated at different time conditions (2, 10, 15 h).

The optical properties of the STO-based materials were investigated by ultraviolet-visible diffuse reflectance spectroscopy (Figure 10).

As expected, STO (1:3) and STO (1:5) had an energy band gap of 3.2 eV in agreement with the literature (Figure S8) [34], which explains the lack of absorption in the visible range (400–700 nm). In contrast, all the Ag-STO samples exhibited an enhancement in the visible light response that arises from the successful double modification. The Ag^+^-doped STO lattice and Ag nanoparticle decoration onto the STO’s surface were in agreement with the XRD results. The Ag^+^ species introduced in the STO’s lattice indicates new electronic energy states forming that lead to a lower energy band gap and, hence, to an efficient charge transfer. The LSPR effect and the Schottky barrier placed at the interface of STO and Ag nanoparticles favor the highly efficient light absorption and charge carrier separation, respectively [18,35]. Figure 10a illustrates a correlation between the Ag weight ratio and the intensity of the response in the visible region. In fact, the 5.8 wt.% and 8.0 wt.% Ag-STO photocatalysts exhibited a slight shift towards the visible light region and the most intense absorption, two crucial features of simultaneous Ag-doped and -decorated materials (e.g., Ag-TiO_2_), indicating a positive influence on the photocatalytic properties of the material. Furthermore, it is worth noting that the LSPR intensity depends upon the surrounding environment, shape, and size distribution (10–100 nm) of Ag nanoparticles [35,36]. Therefore, according to the Rietveld refinement method, the Ag particles that are 5.8 wt.% and 8.0 wt.% Ag-STO photocatalysts measured ≈8 and ≈17 nm, resulting in a broad visible light range.

On the other hand, the findings of the effect of heat treatment conditions on the optical properties of 8.0 wt.% Ag-STO were interesting (Figure 10b). The photocatalyst treated for a longer time (15 h) exhibited the highest visible absorbance response despite the EDX and XRD results, showing a total loss of Ag on the surface. This phenomenon could be associated with the remaining Ag particles characterized by their small size compared to micro-sized Ag particles observed on the materials treated for a shorter duration, displaying a less intense response [35,36].

### 3.2. Photocatalytic Activity

**Figure 11:**
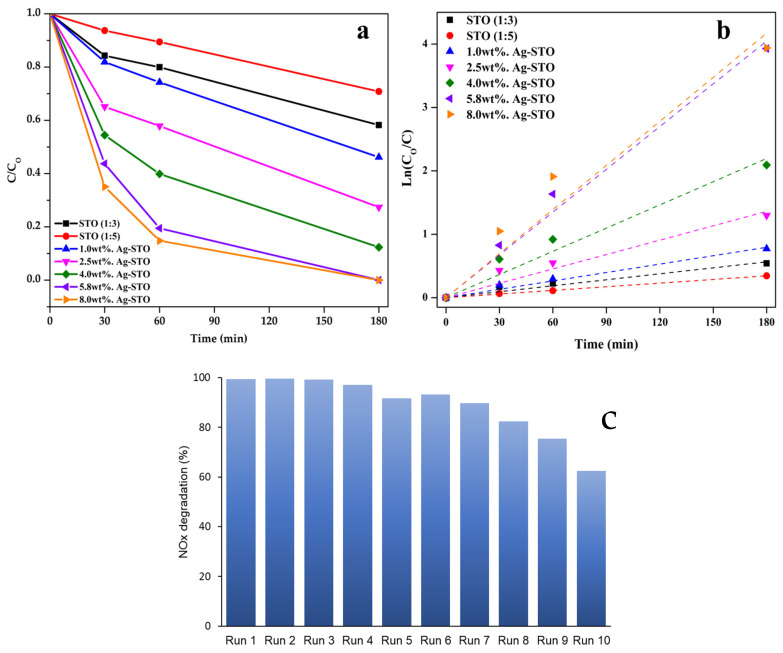
(**a**) NOx photocatalytic removal efficiency reported as C/Co as a function of time, (**b**) photocatalytic kinetic studies of the synthesized STO and Ag-STO photocatalysts, (**c**) reusability of 8.0 wt.% Ag-STO photocatalyst for NOx degradation under LED irradiation.

The photocatalytic activity of STO-based materials was assessed by conducting NOx degradation tests under LED (400–700 nm) for 180 min. The results are plotted in Figure 11. For comparison purposes, the results of the blank test under the same experimental conditions in the absence of photocatalysts were included (Figure S9). The photolysis test gave no results (7% NOx photodegradation), which suggests that light does not influence the photodegradation process significantly.

As expected, bare STO (1:5) was inactive (29% NOx photodegradation) due to its wide energy band gap (Figure 11a). Its performance was similar to the STO synthesized in water reported in our previous work (28% NOx photodegradation) [18]. Interestingly, STO (1:3) achieved 13% more photodegradation in the same time frame (42% NOx photodegradation). The difference could be explained by STO (1:3) porosity. The STO (1:3) photocatalyst exhibits well-defined nanocubes and macropores with an average size of 600 nm, contrasting with the aggregated particles and mesopores observed in STO (1:5) material. Macro/mesoporous materials like STO (1:3) pose the advantages of improved pollutant transport, fewer diffusion limitations, and enhanced light absorption, which boost the photocatalytic performance [37,38].

Furthermore, the Ag-modified STO materials demonstrated a noteworthy improvement in their NOx photocatalytic efficiency compared to STO (1:3), especially by 5.8 wt.% and 8.0 wt.% Ag-STO photocatalysts achieved thorough NOx photocatalytic degradation under LED within 3 h. The combined effect of Ag^+^ doping and Ag decoration on STO promotes an improvement of charge transfer and the separation of photogenerated charge carriers, as well as plasmon resonance effects, resulting in the extension of the light absorption into the visible range [18,35].

To compare our findings with other data available in the literature, Zhang et al. [16] reported the NO photocatalytic degradation by Ag-STO nanocomposites synthesized via a one-pot solvothermal method using a 1:6 water/ethanol volume ratio. The best performing material, 0.5 wt.% Ag-decorated STO, achieved 30% NO degradation under visible light after 30 min. Moreover, the research elucidated that Ag loading higher than 1 wt.% increased the odds of charge carrier recombination, worsening the photocatalytic performance. In contrast, the Ag-STO photocatalysts prepared in this work demonstrated that employing the one-pot sol-gel method with a lower water/ethanol volume ratio and a higher Ag amount, especially with 8.0 wt.%, made it possible to double the NOx photodegradation (65%) within 30 min.

Ma et al. [17] recently reported that 15 mol.%Ag-STO was a promising material, reaching 70% NO photodegradation under visible light within 30 min. It is worth noting that the amount of silver employed by the authors corresponds to 9 wt.% of silver, which is higher than the maximum amount required in the present work. In addition, these results were obtained by running photocatalytic tests using four times the amount of catalyst (200 mg) with respect to that mentioned in Section 2.6. Lastly, the authors employed a hydrothermal method for STO synthesis followed by a photochemical method to obtain the final STO-Ag catalysts. Therefore, the 8.0 wt.% Ag-STO photocatalyst described in this study provides numerous advantages, such as its excellent photocatalytic performance, the minimum amount required per reaction, and the straightforward preparation method suitable for large-scale production, which make it a promising photocatalyst.

The corresponding first-order kinetics plot is illustrated in Figure 11b. It indicates that the 8.0 wt.% Ag-STO and 5.8 wt.% Ag-STO materials exhibited the highest degradation rates (0.023 min^−1^ and 0.022 min^−1^, respectively), which is almost seven times higher than the bare STO (1:3). From a kinetic and an effectiveness point of view, these results suggest more hazardous pollutants will be eliminated in a shorter time frame with the 8.0 wt.% Ag-STO photocatalyst. In our previous work [18], we detailed the synthesis of the 5 wt.% Ag-STO material using a water-based one-pot approach. This photocatalyst was also modified by Ag nanoparticles onto the STO’s surface, as well as the incorporation of Ag^+^ ion within the STO’s surface. Due to the combined effect of both modifications, the latter achieved complete NOx reduction under LED in 3 h, exhibiting a rate of 0.018 min^−1^ and a slightly greater specific surface area (15 m^2^/g). Conversely, a higher NOx degradation rate was obtained by increasing the Ag amount to 5.8 wt.% and 8.0 wt.% (0.0022 min^−1^ and 0.023 min^−1^, respectively), which definitely sped up the photodegradation process. Lastly, concerning the successive photocatalytic reuses (Figure 11c), it is possible to observe a slight decrease in the NOx photocatalytic efficiency of the 8.0 wt.% Ag-STO material after the first four cycles of about 10%. However, after the seventh reuse, the drop-in catalyst activity is more marked, losing 37% of activity at the tenth cycle.

The slow and gradual activity loss can be reasonably attributed to nitrate deposition on the photocatalyst’s surface [18], causing the active sites to be blocked. To confirm this hypothesis, the spent materials were repeatedly washed with small aliquots of water and were subjected to a nitrate colorimetric test with positive results.

XRD analysis before and after the photocatalytic reactions was crucial for assessing the stability of the material’s crystal structure. The XRD pattern observed in Figure S10 suggests the cubic crystal structure of the 8.0 wt.% Ag-STO photocatalyst does not exhibit particular modifications after ten successive photocatalytic reactions, evidencing its high stability and potential, making it a promising alternative for indoor NOx photocatalytic purification.

Lastly, the 8.0 wt.% Ag-STO subjected to 1100 °C was also tested to evaluate its thermal stability at the conditions commonly used in specific industrial processes. The results are reported in Figure S11 and Table 4. The photocatalytic efficiency exhibited by all the samples was correlated with the time required to reach 1100 °C. In fact, the 8.0 wt.% Ag-STO_15h showed the lowest photocatalytic efficiency (30%) attributed to the aggregation of STO particles and the consequent reduction in the surface area (5 m^2^/g), as well as to the reduction in Ag particles exposed to the surface of the catalyst (Figure S5). The 8.0 wt.% Ag-STO_10h and 8.0 wt.% Ag-STO_2h samples achieved 50% and 68% of NOx photocatalytic degradation, respectively, compared to the 8 wt.% Ag-STO photocatalyst calcined at 900 °C. Based on the FE-SEM images (Figure 4 and Figures S3–S5), it can be deduced that longer thermal treatment periods led to fewer amounts of aggregated Ag particles present on the surface of STO. Indeed, no Ag particles were detected in the 8.0 wt.% Ag-STO_1100C_15h photocatalyst. In contrast, the other samples exhibited a considerable presence of agglomerated silver particles on the STO surface. Despite their odd distribution, these particles played a significant role in photocatalytic NOx degradation.

## 4. Conclusions

In the present work, the strontium titanate (STO) photocatalyst was successfully synthesized by a one-pot sol-gel method, assessing different water/ethanol volume ratios. The introduction of ethanol as a solvent, especially at a reduced volume ratio of 1:3, resulted in outstanding improvement, including a larger specific surface area (23 m^2^/g) and the formation of macropores (>600 nm). These structural modifications significantly impacted the photocatalytic degradation towards NOx after 3 h under LED. Building upon these promising results, this eco-friendly, cost-effective, and simple sol-gel approach was tailored to fabricate Ag-STO photocatalysts with different Ag mass ratios. Their NOx photodegradation potential was also comparatively investigated. The Ag-STO sample with 8 wt.% Ag showed the highest photocatalytic rate, achieving complete NOx elimination after 4 h under LED light. The improvement can be ascribed to the large amount of Ag, along with the synergistic effect of a dual modification of the STO material: Ag^+^ doping and Ag- decorated STO. Moreover, in industrial applications where processes can exceed the Ag melting point working temperature (i.e., 1100 °C), an assessment of the 8 wt.% Ag-STO photocatalyst’s thermal stability revealed that by regulating the calcination parameters, it was feasible to preserve its photoactivity partially. Therefore, the 8 wt.% Ag-STO photocatalyst is proposed as a promising and potent material for effective indoor air purification.

## Figures and Tables

**Table 1:** Thermal treatment conditions for 8% Ag-STO photocatalyst calcined at 1100 °C.

Time to Reach the Desired 1100 °C (hours)	Label
2	8.0 wt.% Ag-STO_1100C_2h
10	8.0 wt.% Ag-STO_1100C_10h
15	8.0 wt.% Ag-STO_1100C_15h

**Table 2:** Results of the Rietveld refinements on XRD patterns collected at ID22 beamline of the ESRF.

Sample	5.8 wt.% Ag-STO	8.0 wt.% Ag-STO
**Phase**	**Sr_0.90_Ti_0.02_O_3_**	**Sr_0.86_Ti_0.05_O_3_**
Sp. Group	$Pm\bar{3}m$	$Pm\bar{3}m$
a/Å	3.90870(1)	3.90989(1)
Wt.%	95.12(1)	94.10(1)
**Phase**	**Ag-broad**	**Ag-broad**
Sp. Group	$Fm\bar{3}m$	$Fm\bar{3}m$
a/Å	4.0886(2)	4.0950(2)
Wt.%	1.80	3.62(2)
Dv/nm	8.4	17
**Phase**	**Ag-sharp**	**Ag-sharp**
Sp. Group	$Fm\bar{3}m$	$Fm\bar{3}m$
a/Å	4.09130(2)	4.09207(2)
Wt.%	3.08(1)	2.15(2)
**Wt.% Ag_tot_**	**4.88(4)**	**5.77(4)**
**Phase**	**TiO_2_**	**TiO_2_**
Sp. Group	${P4}_{2}/mnm$	${P4}_{2}/mnm$
a/Å	---------	4.5933(3)
c/Å	---------	2.9603(4)
Wt.%	---------	0.13(1)
U/Å^2^	0.00589(2)	0.00662(2)
Rp	0.0354	0.0362
R(F^2^)	0.0319	0.0306
χ^2^	8.17	9.63

**Table 3:** BET surface area, pore volume, and average size of the obtained photocatalysts.

Photocatalyst	BET Surface (m^2^/g)	Pore Volume (cm^3^/g)
STO (1:3)	23	0.11
STO (1:5)	23	0.15
8%Ag-STO	13	0.11
8 wt.% Ag-STO_1100C_2h	8	0.03
8 wt.% Ag-STO_1100C_10h	10	0.04
8 wt.% Ag-STO_1100C_15h	5	0.03

**Table 4:** First-order rate constants of the prepared photocatalyst for the NOx photocatalytic degradation under LED.

Photocatalyst	Rate Constant (min^−1^)
STO (1:3)	0.003
STO (1:5)	0.002
1.0 wt.% Ag-STO	0.004
2.5 wt.% Ag-STO	0.008
4.0 wt.% Ag-STO	0.012
5.8 wt.% Ag-STO	0.022
8.0 wt.% Ag-STO	0.023
8.0 wt.% Ag-STO_1100C_2h	0.007
8.0 wt.% Ag-STO_1100C_10h	0.005
8.0 wt.% Ag-STO_1100C_15h	0.002

## Data Availability

Data are available upon request.

## Supplementary Materials

The following supporting information can be downloaded at: https://www.mdpi.com/article/10.3390/nano14201663/s1, Figure S1: Rietveld refinements of 1.0% Ag-STO (panel A) and 5.8% Ag-STO (panel B) samples. Experimental XRD patterns, fitted curve, and residuals are depicted as black symbols and red and blue curves, respectively; Figure S2: Zoom of the diffraction peak Ag(111) for the 8.0%wt.Ag-STO photocatalysts exposed for different times at the highest temperature (1100 °C); Figure S3: SEM image (a), elemental mapping of strontium (b), titanium (c), oxygen (d), silver (e), and EDX spectrum (f) of 8.0 wt.% Ag-STO_1100C_2h photocatalyst; Figure S4: SEM image (a), elemental mapping of strontium (b), titanium (c), oxygen (d), silver (e), and EDX spectrum (f) of 8 wt.% Ag-STO_1100C_10h photocatalyst; Figure S5: SEM image (a), elemental mapping of strontium (b), titanium (c), oxygen (d), silver (e), and EDX spectrum (f) of 8 wt.% Ag-STO_1100C_15h photocatalyst; Figure S6: SEM image (a), elemental mapping of strontium (b), titanium (c), oxygen (d), carbon (e), and EDX spectrum (f) of STO (1:5) photocatalyst; Figure S7: N_2_ adsorption/desorption isotherms at −196 °C; Figure S8: Tauc plot of Ag-STO photocatalysts at 900 °C; Figure S9: Photolysis study for NOx degradation under LED light without any catalyst; Figure S10: XRD pattern of the 8.0 wt.% Ag-STO photocatalyst (a) before and (b) after ten successive photocatalytic reactions; Figure S11: NOx photocatalytic removal efficiency C/Co as a function of time and (b) first-order kinetic plot of 8.0 wt.% Ag-STO photocatalyst calcined at 1100 °C with different heat treatment conditions; Table S1. Theoretical molar ratios between elements in Ag-STO samples; Table S2: Structural parameters extracted from Rietveld refinements of the Miniflex measurements; Table S3: Structural parameters extracted from Rietveld refinements of the Miniflex measurements.

## References

[1] Bălă G.P., Râjnoveanu R.M., Tudorache E., Motișan R., Oancea C. (2021). Air pollution exposure—The (in)visible risk factor for respiratory diseases. Environ. Sci. Pollut. Res..

[2] Sicard P., Agathokleous E., Anenberg S.C., De Marco A., Paoletti E., Calatayud V. (2023). Trends in urban air pollution over the last two decades: A global perspective. Sci. Total Environ..

[3] Lasek J., Yu Y.H., Wu J.C.S. (2013). Removal of NOx by photocatalytic processes. J. Photochem. Photobiol. C Photochem. Rev..

[4] Panigrahi T.H., Sahoo S.R., Murmu G., Maity D., Saha S. (2022). Current challenges and developments of inorganic/organic materials for the abatement of toxic nitrogen oxides (NOx)—A critical review. Prog. Solid State Chem..

[5] Talaiekhozani A., Rezania S., Kim K.H., Sanaye R., Amani A.M. (2021). Recent advances in photocatalytic removal of organic and inorganic pollutants in air. J. Clean. Prod..

[6] Serpone N. (2018). Heterogeneous photocatalysis and prospects of TiO_2_-based photocatalytic DeNOxing the atmospheric environment. Catalysts.

[7] Pietrogrande M.C., Casari L., Demaria G., Russo M. (2021). Indoor air quality in domestic environments during periods close to italian covid-19 lockdown. Int. J. Environ. Res. Public Health.

[8] Hanaor D.A.H., Sorrell C.C. (2011). Review of the anatase to rutile phase transformation. J. Mater. Sci..

[9] Tilley R.J.D. (2017). Perovskites: Structure–Property Relationship. MRS Bull..

[10] Kumar A., Kumar A., Krishnan V. (2020). Perovskite Oxide Based Materials for Energy and Environment-Oriented Photocatalysis. ACS Catal..

[11] Wang W., Tadé M.O., Shao Z. (2015). Research progress of perovskite materials in photocatalysis- and photovoltaics-related energy conversion and environmental treatment. Chem. Soc. Rev..

[12] Phoon B.L., Lai C.W., Juan J.C., Show P.L., Pan G.T. (2019). Recent developments of strontium titanate for photocatalytic water splitting application. Int. J. Hydrogen Energy.

[13] Varghese R., Sreekala C.O., Kurian S., Thomas J.K. (2023). Insight into structural, optical, electrical, dielectric, and photovoltaic behaviour of cerium-doped strontium titanate by a modified combustion method. J. Mater. Sci. Mater. Electron..

[14] Haghshenas N., Falletta E., Cerrato G., Giordana A., Bianchi C.L. (2023). Tuning the visible-light-driven photocatalytic properties of multi-decorated TiO_2_ by noble metals towards both propionic acid and NOx degradation. Catal. Commun..

[15] Zhou N., López-Puente V., Wang Q., Polavarapu L., Pastoriza-Santos I., Xu Q.H. (2015). Plasmon-enhanced light harvesting: Applications in enhanced photocatalysis, photodynamic therapy and photovoltaics. RSC Adv..

[16] Zhang Q., Huang Y., Xu L., Cao J.J., Ho W., Lee S.C. (2016). Visible-Light-Active Plasmonic Ag-SrTiO_3_ Nanocomposites for the Degradation of NO in Air with High Selectivity. ACS Appl. Mater. Interfaces.

[17] Ma H., Yang W., Tang H., Pan Y., Li W., Fang R., Shen Y., Dong F. (2023). Enhance the stability of oxygen vacancies in SrTiO_3_ via metallic Ag modification for efficient and durable photocatalytic NO abatement. J. Hazard. Mater..

[18] Ordoñez M.F., Cerrato G., Giordana A., Di Michele A., Falletta E., Bianchi C.L. (2023). One-pot synthesis of Ag-modified SrTiO_3_: Synergistic effect of decoration and doping for highly efficient photocatalytic NOx degradation under LED. J. Environ. Chem. Eng..

[19] Aravinthkumar K., Praveen E., Jacquline Regina Mary A., Raja Mohan C. (2022). Investigation on SrTiO_3_ nanoparticles as a photocatalyst for enhanced photocatalytic activity and photovoltaic applications. Inorg. Chem. Commun..

[20] Fitch A., Dejoie C., Covacci E., Confalonieri G., Grendal O., Claustre L., Guillou P., Kieffer J., de Nolf W., Petitdemange S. (2023). ID22—The high-resolution powder-diffraction beamline at ESRF. J. Synchrotron Radiat..

[21] Larson A.C., Von Dreele R.B. *General Structure Analysis System (GSAS)*; Los Alamos National Laboratory Report LAUR 2000. 86. https://www.scirp.org/reference/referencespapers?referenceid=105526.

[22] Toby B.H. (2001). EXPGUI, a graphical user interface for GSAS. J. Appl. Crystallogr..

[23] Williamson G.K., Hall W.H. (1953). X-ray line broadening from filed aluminium and wolframL’elargissement des raies de rayons x obtenues des limailles d’aluminium et de tungsteneDie verbreiterung der roentgeninterferenzlinien von aluminium- und wolframspaenen. Acta Metall..

[24] Landi S., Segundo I.R., Freitas E., Vasilevskiy M., Carneiro J., Tavares C.J. (2022). Use and misuse of the Kubelka-Munk function to obtain the band gap energy from diffuse reflectance measurements. Solid State Commun..

[25] Kuang Q., Yang S. (2013). Template synthesis of single-crystal-like porous SrTiO_3_ nanocube assemblies and their enhanced photocatalytic hydrogen evolution. ACS Appl. Mater. Interfaces.

[26] Hui Q., Tucker M.G., Dove M.T., Well S.A.A., Keen D.A. (2005). Total scattering and reverse Monte Carlo study of the 105 K displacive phase transition in strontium titanate. Phys. Condens. Matter.

[27] Cabassi R., Checchia S., Trevisi G., Scavini M. (2021). Low Temperature Ferroelectricity in Strontium Titanate Domain Walls Detected by Depolarization Pyrocurrents. Mater. Today Commun..

[28] Howard C.J., Sabine T.M., Dickson F. (1991). Structural and Thermal Parameters for Rutile and Anatase. Acta Crystallogr. B.

[29] Wyckoff R.W.G. (1963). Crystal Structures.

[30] Wei X., Xu G., Ren Z., Xu C., Shen G., Han G. (2008). PVA-assisted hydrothermal synthesis of SrTiO_3_ nanoparticles with enhanced photocatalytic activity for degradation of RhB. J. Am. Ceram. Soc..

[31] He X., Yang Y., Li Y., Chen J., Yang S., Liu R., Xu Z. (2022). Effects of structure and surface properties on the performance of ZnO towards photocatalytic degradation of methylene blue. Appl. Surf. Sci..

[32] Sotomayor F.J., Cychosz K.A., Thommes M. (2018). Characterization of Micro/Mesoporous Materials by Physisorption: Concepts and Case Studies. Acc. Mater. Surf. Res..

[33] Sharma N., Hernadi K. (2022). The Emerging Career of Strontium Titanates in Photocatalytic Applications: A Review. Catalysts.

[34] Wu Z., Zhang Y., Wang X., Zou Z. (2017). Ag@SrTiO_3_ nanocomposite for super photocatalytic degradation of organic dye and catalytic reduction of 4-nitrophenol. New J. Chem..

[35] Varma R.S., Thorat N., Fernandes R., Kothari D.C., Patel N., Miotello A. (2016). Dependence of photocatalysis on charge carrier separation in Ag-doped and decorated TiO_2_ nanocomposites. Catal. Sci. Technol..

[36] Zhang X., Chen Y.L., Liu R.-S., Tsai D.P. (2013). Plasmonic photocatalysis. Rep. Prog. Phys..

[37] Sun T., Shan N., Xu L., Wang J., CHen J., Zakhidov A.A., Baughman R.H. (2018). General Synthesis of 3D Ordered Macro-/Mesoporous Materials by Templating Mesoporous Silica Confined in Opals. Chem. Mater..

[38] Arutanti O., Nandiyanto A.B.D., Ogi T., Kim T.O., Okuyama K. (2015). Influences of porous structurization and pt addition on the improvement of photocatalytic performance of WO_3_ particles. ACS Appl. Mater. Interfaces.

