# Supplementary material for: Investigating the Synergistic Effect of Decoration and Doping in Silver/Strontium Titanate for Air Remediation
Source: Nanomaterials (Basel). 2024 Oct 16;14(20):1663. doi: 10.3390/nano14201663 (PMC11510593; doi:10.3390/nano14201663)
Supplement: Supplementary file 1 [file nanomaterials-14-01663-s001.zip › nanomaterials-3243135-supplementary.pdf]

Investigating the Synergistic Effect of Decoration and Doping in Silver/Strontium Titanate for Air Remediation

Marcela Frías Ordóñez [1,2], Elisabetta Sacco [1], Marco Scavini [1], Giuseppina Cerrato [2,3], Alessia Giordana [2,3], Ermelinda Falletta [1,2,*] and Claudia Letizia Bianchi [1,2]

[1] Department of Chemistry, University of Milan, Via Golgi 19, 20133 Milano, Italy; marcela.frias@unimi.it (M.F.O.); elisabetta.sacco@unimi.it (E.S.); marco.scavini@unimi.it (M.S.); ermelinda.falletta@unimi.it (E.F.); claudia.bianchi@unimi.it (C.L.B.); ermelinda.falletta@unimi.it (E.F.)
[2] Consorzio Interuniversitario Nazionale per la Scienza e Tecnologia dei Materiali (INSTM), Via Giusti 9, 50121 Florence, Italy; giuseppina.cerrato@unito.it (G.C.); alessia.giordana@unito.it (A.G.); ermelinda.falletta@unimi.it (E.F.); claduia.bianchi@unimi.it (C.L.B.)
[3] Department of Chemistry, University of Turin, Via Giuria 7, 10125 Turin, Italy
* Correspondence: ermelinda.falletta@unimi.it

Table S1. Theoretical molar ratios between elements in Ag-STO samples

Wt.% Ag	nSr	nAg	nTi
1	0.98	0.02	1
2.5	0.96	0.04	1
4	0.93	0.07	1
5.8	0.90	0.10	1
8	0.86	0.14	1

Table S2. Ag-STO samples structural parameters extracted from Rietveld refinements of the Miniflex measurements

**Sample**	**STO (1:3)**	**1.0 wt.% Ag-STO**	**2.5 wt.% Ag-STO**	**4.0 wt.% Ag-STO**	**5.8 wt.% Ag-STO**	**8.0 wt.% Ag-STO**
**Phase**	**$SrTiO_3$**	**$SrTiO_3$**	**$SrTiO_3$**	**SrTiO**	**$SrTiO_3$**	**$SrTiO_3$**
Sp. Group	$Pm\bar{3}m$	$Pm\bar{3}m$	$Pm\bar{3}m$	$Pm\bar{3}m$	$Pm\bar{3}m$	$Pm\bar{3}m$
a/Å	3.90712(4)	3.90729(9)	3.90536(6)	3.90870(2)	3.90721(4)	3.90769(4)
Wt.%	94.5(1)	94.1(1)	98.7(1)	97.6(1)	96.1(1)	95.1(1)

Phase	Ag	Ag	Ag	Ag	Ag	Ag
Sp. Group	$Fm\bar{3}m$	$Fm\bar{3}m$	$Fm\bar{3}m$	$Fm\bar{3}m$	$Fm\bar{3}m$	$Fm\bar{3}m$
a/Å	-------	4.0867(3)	4.0874(2)	4.0874(2)	4.0887(1)	4.0900(1)
Wt.%	-------	0.62(2)	1.3(1)	2.4(1)	3.9(1)	4.9(1)
**Phase**	**TiO_2**	**TiO_2**	**TiO_2**	**TiO_2**	**TiO_2**	**TiO_2**
Sp. Group	$I4_1/amd$	$I4_1/amd$	$I4_1/amd$	$I4_1/amd$	$I4_1/amd$	$I4_1/amd$
a/Å	3.758(2)	3.733(3)	-------	-------	-------	-------
c/Å	9.943(8)	10.07(1)	-------	-------	-------	-------
Wt.%	5.5(1)	5.3(1)	-------	-------	-------	-------
U/Å^2	0.0055(2)	-0.0053(9)	0.0040(3)	0.151(3)	0.0037(1)	0.0034(1)
Rp	0.0305	0.0524	0.0497	0.0471	0.0239	0.0236
R(F^2)	0.0487	0.0566	0.0406	0.0388	0.0353	0.0356

Table S3. Structural parameters extracted from Rietveld refinements of XRD patterns collected on the 8.0 wt.% Ag-STO samples using the Miniflex measurements

**Sample**					
**Phase**	**$SrTiO_3$**	**$SrTiO_3$**	**$SrTiO_3$**	**$SrTiO_3$**	**$SrTiO_3$**
**Treatment**	**900 °C**	**1100 °C 15 h**	**1000 °C 10 h**	**1100 °C 10 h**	**1100 °C 2 h**
Sp. Group	$Pm\bar{3}m$	$Pm\bar{3}m$	$Pm\bar{3}m$	$Pm\bar{3}m$	$Pm\bar{3}m$
a/Å	3.90769(4)	3.90698(6)	3.90781(4)	3.90688(5)	3.90645(6)
Wt.%	95.1(1)	97.3(1)	95.2(1)	96.3(1)	95.8(1)
**Phase**	**Ag**	**Ag**	**Ag**	**Ag**	**Ag**
Sp. Group	$Fm\bar{3}m$	$Fm\bar{3}m$	$Fm\bar{3}m$	$Fm\bar{3}m$	$Fm\bar{3}m$
a/Å	4.0900(1)	4.0870(1)	4.0886(1)	4.0875(1)	4.0879(1)
Wt.%	4.9(1)	2.7(1)	4.8(1)	3.6(1)	4.2(1)
U/$Å^2$	0.0034(1)	-0.0029(3)	0.0028(2)	-0.0054(3)	0.0010(3)
Rp	0.0236	0.0254	0.0198	0.0260	0.0485
$R(F^2)$	0.0356	0.0370	0.0395	0.0642	0.0469

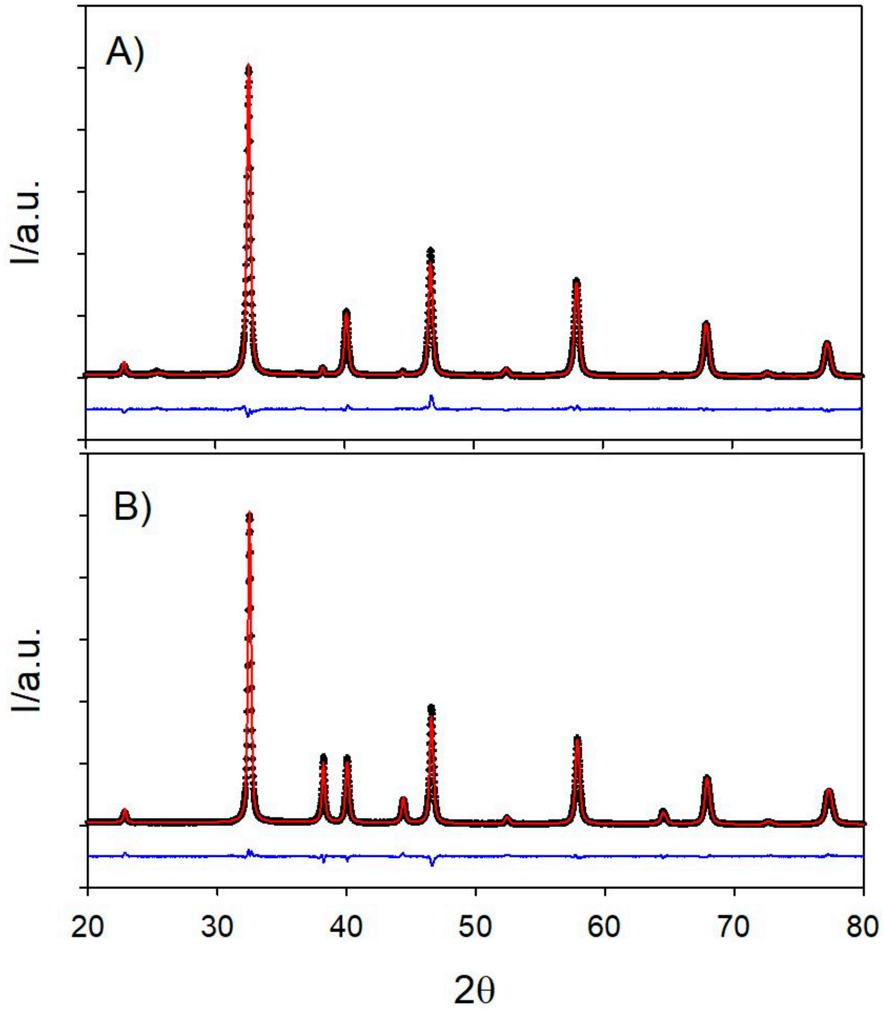

Figure S1. Rietveld refinements of 1.0% Ag-STO (panel A) and 5.8% Ag-STO (panel B) samples. Experimental XRPD patterns, fitted curve and residuals are depicted as black symbols, red and blue curves, respectively.

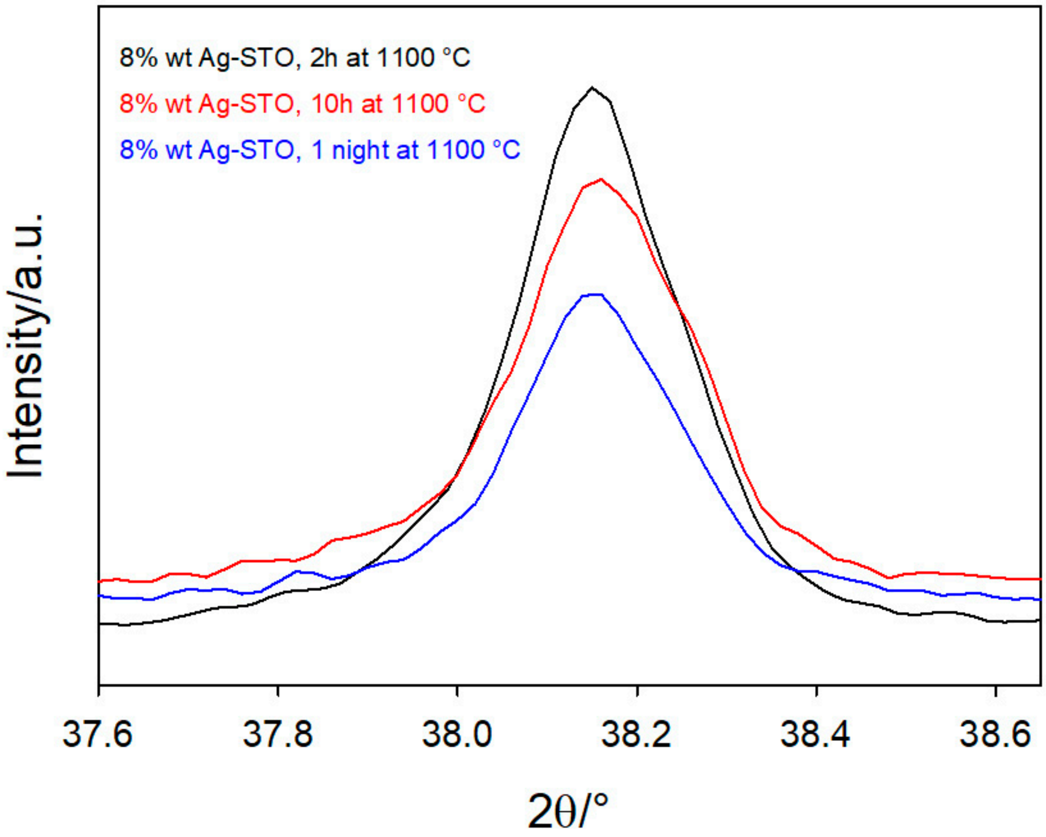

Figure S2. Zoom of the diffraction peak Ag(111) for the 8.0%wt.Ag-STO photocatalysts exposed for different times at the highest temperature (1100 °C).

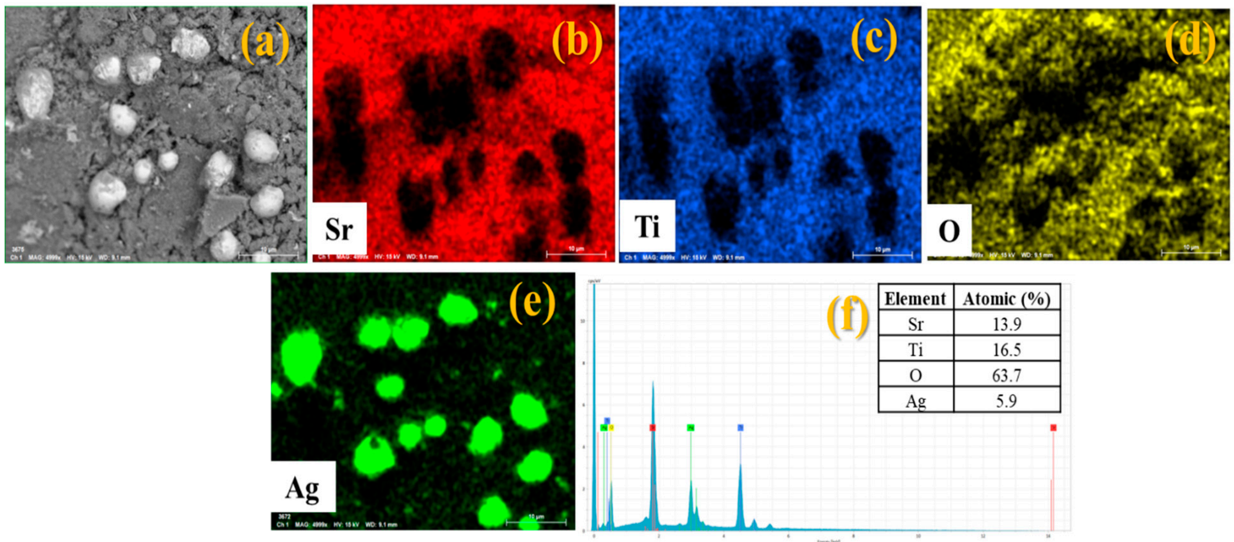

Figure S3. SEM image (a), elemental mapping of Strontium (b), Titanium (c), Oxygen (d), Silver (e), and EDX spectrum (f) of 8.0 wt.% Ag-STO_1100C_2h photocatalyst.

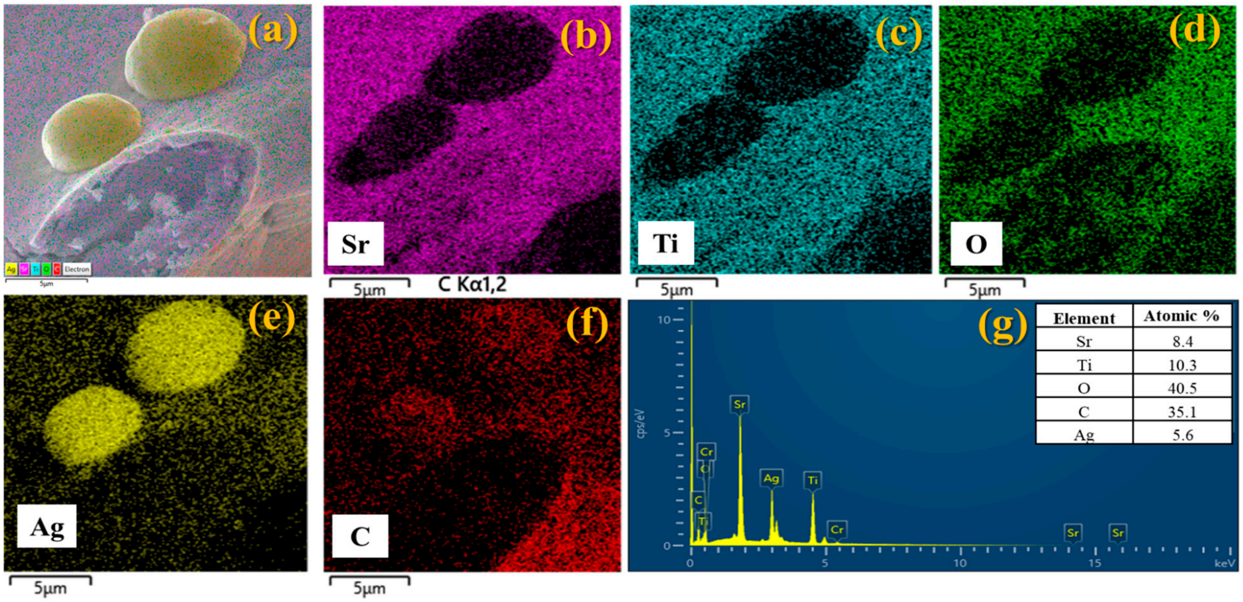

Figure S4. SEM image (a), elemental mapping of Strontium (b), Titanium (c), Oxygen (d), Silver (e), Carbon (f), and EDX spectrum (g) of 8 wt.% Ag-STO_1100C_10h photocatalyst.

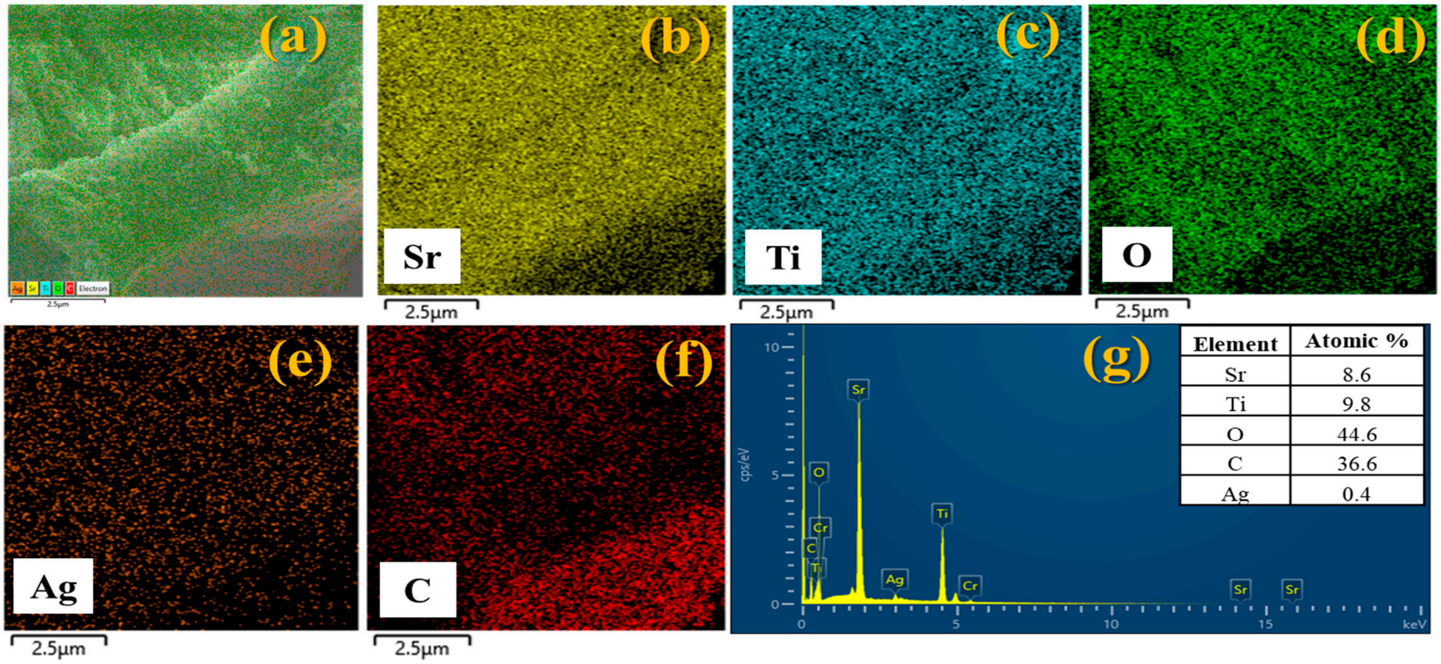

Figure S5. SEM image (a), elemental mapping of Strontium (b), Titanium (c), Oxygen (d), Silver (e), and EDX spectrum (f) of 8 wt.% Ag-STO_1100C_15h photocatalyst.

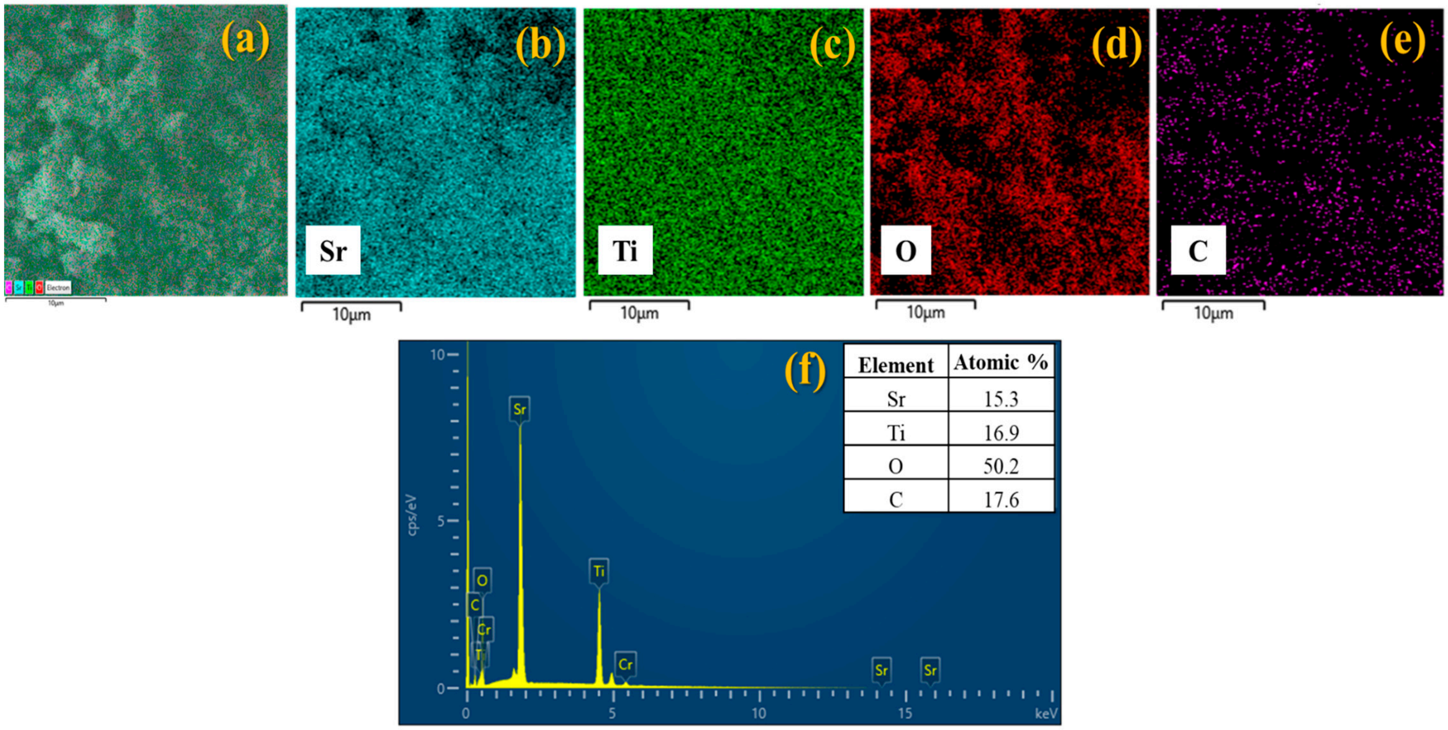

Figure S6. SEM image (a), elemental mapping of Strontium (b), Titanium (c), Oxygen (d), Carbon (e), and EDX spectrum (f) of STO (1:5) photocatalyst.

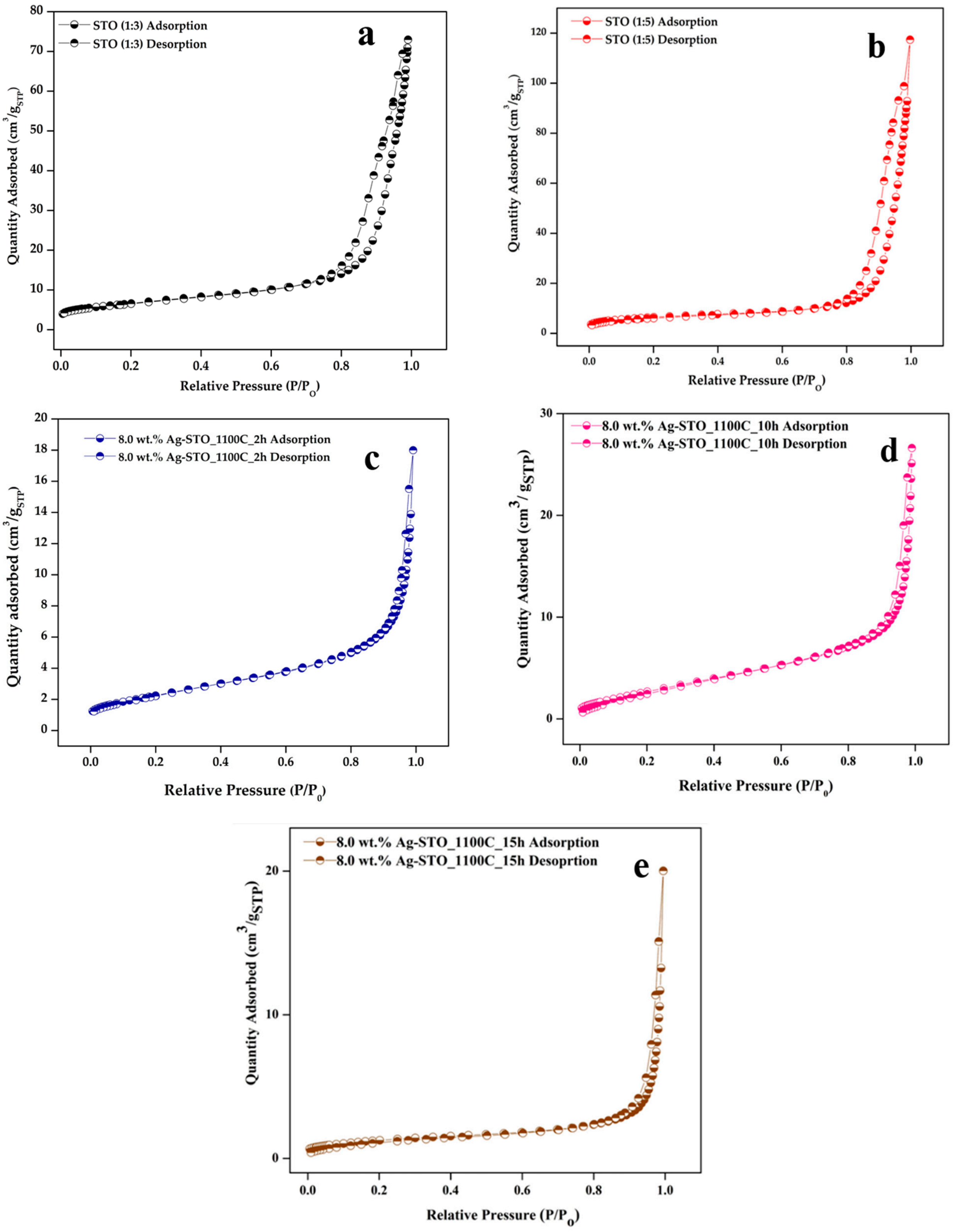

Figure S7. N_2 adsorption/desorption isotherms at -196°C;

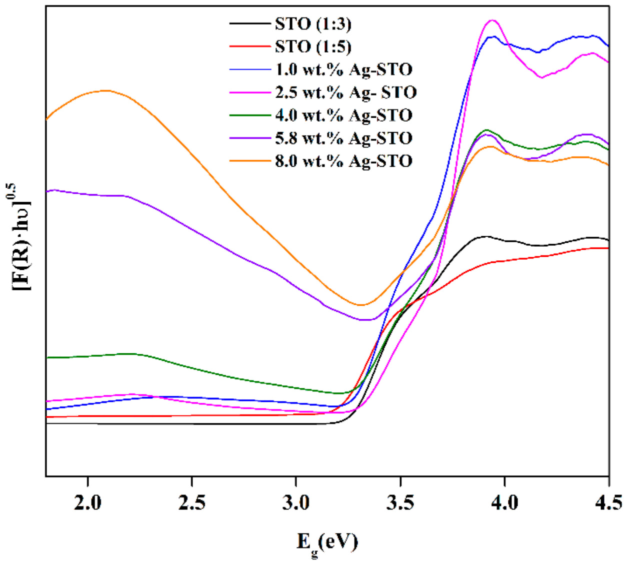

Figure S8. Tauc Plot of Ag-STO photocatalysts at 900ºC.

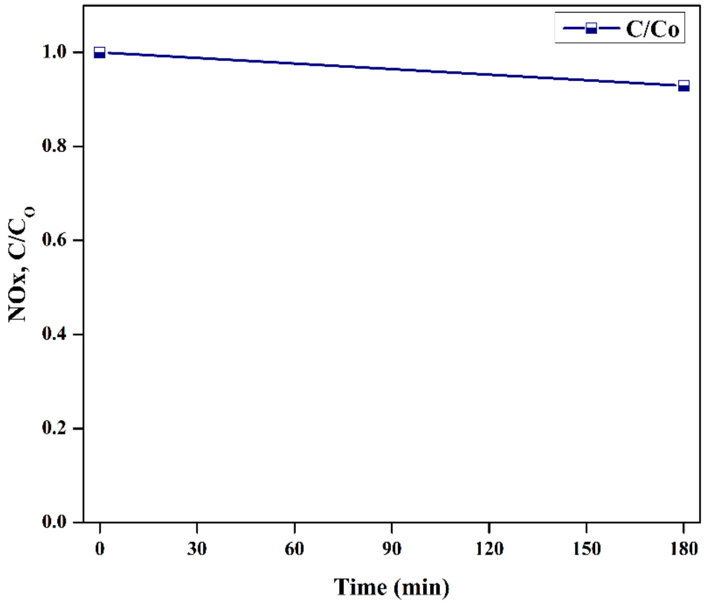

Figure S9. Photolysis study for NOx degradation under LED light without any catalyst.

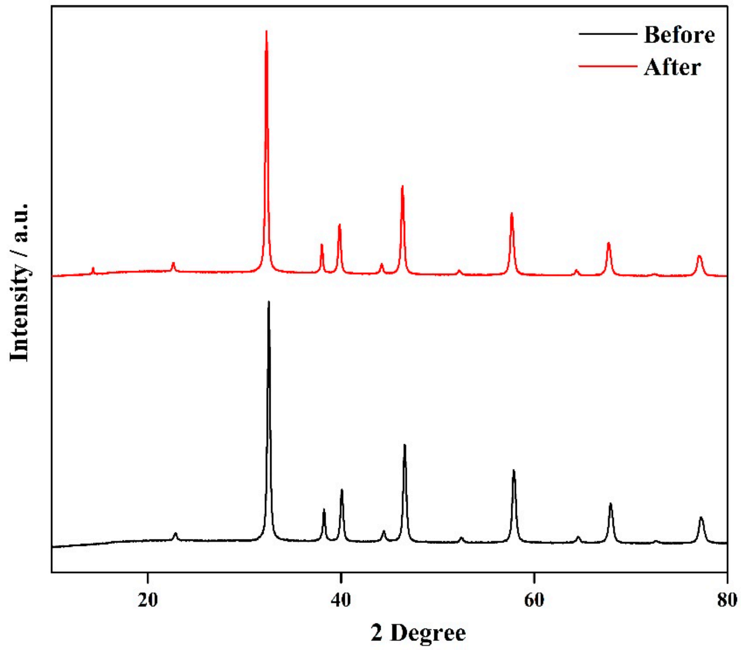

Figure S10. XRPD pattern of the 8.0 wt.% Ag-STO photocatalyst (a) before and (b) after ten successive photocatalytic reactions.

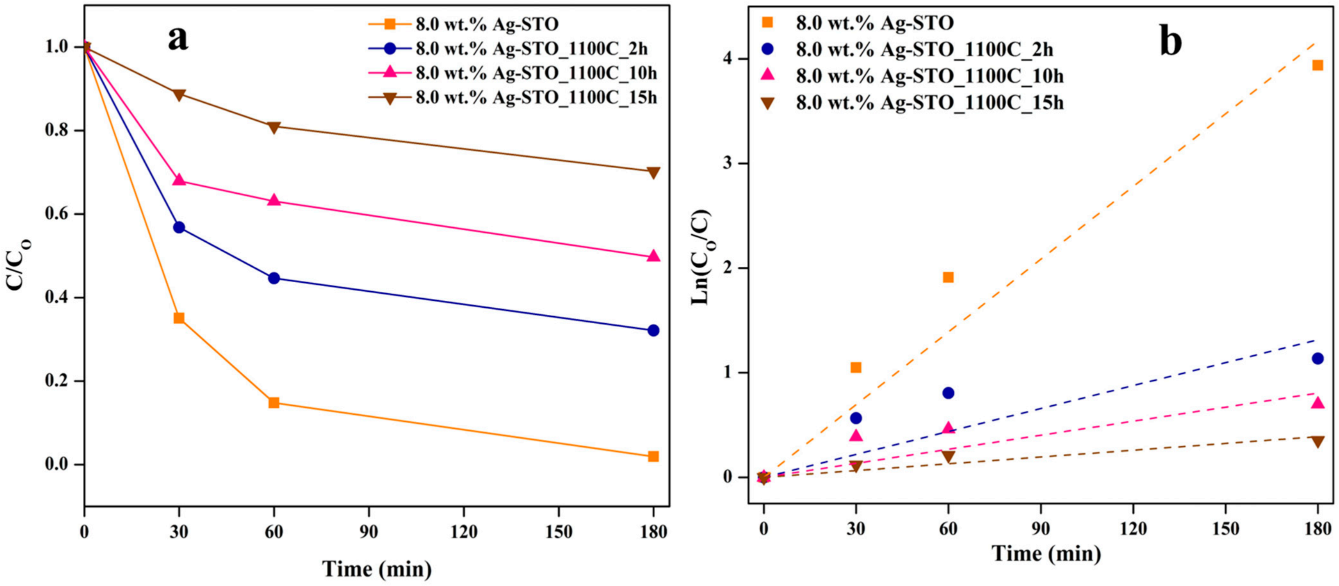

Figure S11. NOx photocatalytic removal efficiency C/Co as a function of time and (b) First-order kinetic plot of 8.0 wt.% Ag-STO photocatalyst calcined at 1100ºC with different heat treatment conditions.
